# Nanoparticle-Based Therapies for Turning Cold Tumors Hot: How to Treat an Immunosuppressive Tumor Microenvironment

**DOI:** 10.3389/fbioe.2021.689245

**Published:** 2021-06-02

**Authors:** Giulio Giustarini, Andrea Pavesi, Giulia Adriani

**Affiliations:** ^1^Singapore Immunology Network (SIgN), Agency for Science, Technology and Research (A^∗^STAR), Singapore, Singapore; ^2^Institute of Molecular and Cell Biology (IMCB), Agency for Science, Technology, and Research (A^∗^STAR), Singapore, Singapore; ^3^Department of Biomedical Engineering, National University of Singapore, Singapore, Singapore

**Keywords:** nanotechnologies, cold tumors, hot tumors, nanoparticles, cancer therapies, tumor immune microenvironment, drug delivery, immunotherapies

## Abstract

Nanotechnologies are rapidly increasing their role in immuno-oncology in line with the need for novel therapeutic strategies to treat patients unresponsive to chemotherapies and immunotherapies. The tumor immune microenvironment (TIME) has emerged as critical for tumor classification and patient stratification to design better treatments. Notably, the tumor infiltration of effector T cells plays a crucial role in antitumor responses and has been identified as the primary parameter to define hot, immunosuppressed, excluded, and cold tumors. Organic and inorganic nanoparticles (NPs) have been applied as carriers of new targeted therapies to turn cold or altered (i.e., immunosuppressed or excluded) tumors into more therapeutically responsive hot tumors. This mini-review discusses the significant advances in NP-based approaches to turn immunologically cold tumors into hot ones.

## Introduction

Over the last decades, scientists have made a tremendous effort to design new nanotechnologies to fight cancer. Nanotechnologies represent a promising therapeutic tool to reduce cancer’s social and economic burden, which remains a dreadful cause of death worldwide. Various nanotechnologies have demonstrated an improved therapeutic outcome over existing antitumor therapies and made possible targeted therapies by significantly reducing their systemic toxicity (Phuengkham et al., 2019).

Tumors are generally characterized by mutated cells with an uncontrolled proliferation that rapidly lump together with immune, endothelial and mesenchymal cells, and their extracellular matrix, creating a dedicated tumor immune microenvironment (TIME). Recent advances in cancer immunology revealed that specific traits of the TIME correlate with the efficacy of chemotherapy and immunotherapies (Han et al., 2020; Liang et al., 2020). Based on their “immune contexture,” tumors have been categorized as hot, immunosuppressed, excluded, and cold (Galon et al., 2006; Galon and Bruni, 2019). Hot tumors are characterized by (i) a higher infiltration of T cells within the tumor and at the invasive margin, (ii) a higher level of proinflammatory cytokines, and (iii) a better response to immune-checkpoint blockade (ICB) therapies compared to excluded, immunosuppressed and cold tumors. For patient stratification, a standardized scoring system called Immunoscore has been introduced based on the quantification of CD3^+^ lymphocyte populations and ranging between 0 (hot immune tumors) and 4 (cold immune tumors) (Pagès et al., 2018).

Several features of hot tumors participate in the regulation of an antigen-specific response via the expression of inhibitory signals such as the activation of programmed cell death protein 1 (PD-1), cytotoxic T-lymphocyte-associated protein 4 (CTLA4) and other immune checkpoints, and the extracellular-potassium-mediated T cell suppression (Walunas et al., 1994; Ahmadzadeh et al., 2009; Eil et al., 2016). Peculiar driver and passenger mutations in cancer cells also contribute to the great heterogeneity in the TIME (Yoshida et al., 2016; McFarland et al., 2017). Further, physical barriers such as a dense extracellular matrix (ECM) (Heldin et al., 2004) and tumor-associated vasculature, cellular and humoral immunosuppressive components are all key players modulating the TIME (Joyce and Fearon, 2015). The presence of cytokines that regulate the function of cytotoxic CD8^+^ T cells, such as interleukin 15 (IL-15), can influence CD8^+^ T cell presence and persistence within the TIME (Mlecnik et al., 2014).

**FIGURE 1:**
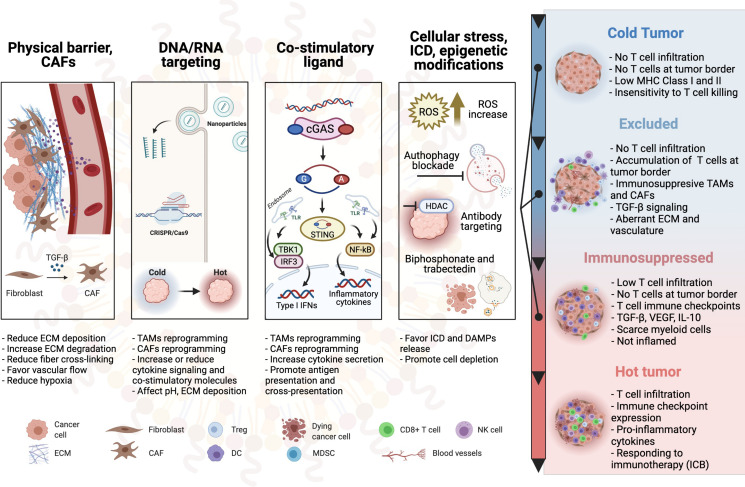
Schematic overview of the nanoparticle-based therapies to turn cold tumors hot. CAF, cancer-associated fibroblast; ECM, extracellular matrix; TAM, tumor-associated macrophage; Treg, regulatory T cell; DC, dendritic cell; TLR, toll-like receptor; MDSC, myeloid-derived suppressor cell; ICD, immunogenic cell death; NK cell, natural killer cell; MHC, major histocompatibility complex; ICB, immune checkpoint blockade.

In this review, we aim to summarize the most recent antitumor strategies that use nanotechnologies to favorably alter the immunosuppressive nature of the TIME, changing immune-defective tumors (cold) into immunogenic and immunoreactive environments (hot) as schematically depicted in Figure 1.

## Nanotechnologies for Therapeutic Delivery

Nanotechnology-enabled drug delivery approaches have been designed and formulated for overcoming major bottlenecks of chemotherapies and immunotherapies. Organic and inorganic nanocarriers have improved tumor targeting and preserved chemical or biological payloads from degradation. Since nanoparticles (NPs) can be recognized and internalized by the mononuclear phagocyte system (MPS), some NPs leverage their uptake by immune cells to reach and accumulate within the tumor site, also referred to as the Trojan horse method (Levy et al., 2016; Qi Y. et al., 2021). Other NPs take advantage of tumor features such as the enhanced permeability and retention (EPR) effect and the lack of lymphatic drainage that promotes small NPs accumulation into the tumor rather than in healthy tissues. In addition to these anatomopathological features of cancer, NPs are often functionalized with specific molecules to promote infiltration and retention within the TIME and cellular targeting (Grobmyer and Moudgil, 2013; Nichols and Bae, 2014). Liposome (Aronson et al., 2021), micelles (Otsuka et al., 2003), gold nanoparticle (Gerosa et al., 2020), carbon nanotubes (Faraji Dizaji et al., 2020), silica/silicon NPs (Adriani et al., 2012; van de Ven et al., 2012), nanorods (Ali et al., 2017), dendrimers (Huang et al., 2020), polymer NPs (Avramovi et al., 2020) are some of the nanotechnologies most utilized for antitumor therapeutic strategies.

### NP-Mediated Shaping of Physical Barriers and Target of Stromal Cells

Tumor immune microenvironment is usually characterized by physical barriers impeding immune cells and therapies to reach the tumor site. Since the penetration of immune cells into the tumor positively correlates with relapse-free survival and immunotherapies efficacy in preclinical and clinical studies, various strategies aiming to reduce physical barriers have been investigated. The predominant physical barrier within the TIME is the ECM (Winkler et al., 2020). Tumor ECM proteins are mainly secreted by abundantly recruited cancer-associated fibroblasts (CAFs) and tumor-associated macrophages (TAMs) (Casey et al., 2009; Afik et al., 2016), although tumor cells participate in ECM proteins deposition (Naba et al., 2012). Nanotechnology-based therapies able to change the density, organization, composition, and post-translational modification of the produced ECM proteins have been developed. Tumor ECM directly regulates effector functions of T cells (Parekh et al., 2005; Leclerc et al., 2019) and induces hypoxia. Hypoxia indirectly regulates the immune response and cell invasion into the tumor by increasing regulatory cytokines (IL-10, TGF-β) and decreasing cell adhesion molecules on tumor-associated blood vessels (Henke et al., 2020).

Two main strategies have been conceived to modulate the ECM promoting a better infiltration of immune cells and therapies within the TIME.

The first strategy aims to reduce the protein deposition or regulate their organization. This strategy was implemented in two *in vivo* studies targeting CAFs. The first study considered photosensitizer-loaded NPs combined with phototherapy for targeted CAF depletion. In contrast, the other study performed CAF reprogramming via gold NPs delivering siRNA against heat shock protein 47 (HSP47, a collagen-specific molecular chaperone) combined with all-*trans* retinoic acid to turn CAFs quiescent. Both studies showed a reduced ECM deposition allowing a better tumor penetration of CD8^+^ T cells and therapeutic NPs (Zhen et al., 2017; Han et al., 2018). Interestingly, Zhao et al. showed in a xenograft mouse model of colorectal cancer that gold NPs possess an intrinsic capacity to decrease ECM protein production (Zhao et al., 2018). Organization of the collagen fibers by post-translational modifications determines tumor stiffness. Administration of NPs coated with an inhibitory antibody against the collagen-cross linking enzyme lysyl oxidase was associated with a more significant suppressive effect of tumor growth than the inhibitory antibody alone (Kanapathipillai et al., 2012).

The second strategy to remodel tumor ECM relies on the metabolic activities of exogenous and endogenous proteases against ECM proteins such as collagen, hyaluronic acid, and fibronectin. Several NPs such as silver/gold particles, micelles, and liposomes (Abdolahinia et al., 2019; Zinger et al., 2019; Huang et al., 2021) have been tested as proteases carriers in different *in vivo* tumor models. Although these existing studies are unanimous in presenting a better penetration into the tumor when the NPs are coated with proteases (Zhou et al., 2016; Xu et al., 2020), none of them showed the effects of proteases on immune cell infiltration.

Granting that the approaches of remodeling tumor ECM to turn a cold tumor hot via NP-based therapies demonstrated therapeutic potential, they still present critical clinical limitations. For example, degradation of collagen could lead to cytokine release, which might sustain inflammatory processes promoting tumorigenesis. Additionally, loss of the containment function of the ECM might facilitate access to the bloodstream and consequent tumor metastasis, leading to a worse prognosis (Bonnans et al., 2014; Fang et al., 2014). For this reason, a deeper validation is required before the effective translation of these strategies to clinical settings.

### NP-Mediated Targeting of Co-inhibitory and Co-stimulatory Ligands/Receptors

To reverse the unfavorable immune composition of cold tumors, NPs have been equipped with molecules capable of either stimulating the immunity against the tumor or inhibiting the regulatory mechanisms that prevent the immune system from fighting cancer cells. A fine-tuning of stimulatory and inhibitory signals from and to the innate immune system is critical to establish a favorable response against the tumor.

Although immune checkpoint blockade (ICB) therapies are an undoubtful step forward in the fight against cancer, they might not reach the desired effectiveness in cold tumors due to the presence of cytokines inhibiting both maturation and activity of antigen-presenting cells, especially dendritic cells (DCs), and inducing polarization of TAMs (Rodell et al., 2018). Immunostimulants or inhibitors of regulatory mechanisms also found applications in cancer vaccine development to boost the presentation of the epitopes or their related mRNA (Ritchie and Li, 2020).

#### Toll-Like Receptors Agonists

Toll-like receptors (TLRs) are Type I transmembrane glycoproteins expressed by cells of the innate immune system and malignant cells. Activation of TLRs leads to the production of proinflammatory cytokines such as type I interferons that mediate an adaptive immune response through activation of DCs. TLRs can be classified into two subgroups according to the type of ligands they respond to and their cellular localization: TLRs recognizing mainly bacterial components located on the plasma membrane such as TLR1/2/4/5/6 and TLRs recognizing DNA and RNA such as TLR3/7/8/9 which are instead located intracellularly on the endosome (Rakoff-Nahoum and Medzhitov, 2009).

Toll-like receptor stimulation may play a role in tumor development via either a protumor or antitumor effect on the mutated cells. In different settings, TLR stimulation was shown to cause either an antitumor effect by directly inducing gene-related programmed cell death or, conversely, to have a protumor effect promoting cell proliferation, mutagenesis, invasion, and even resistance to apoptosis in malignant cells mainly via activation of NF-kB, MAPK cascades and PI3K-Akt pathway (Cen et al., 2018; Kabelitz et al., 2019). Antitumor therapy with nanotechnologies delivering TLR agonists directly to antigen-presenting cells (APCs) prevents TLR degradation and potential side effects of the therapy (Berraondo et al., 2019; Ding et al., 2020; Flórez-Álvarez et al., 2020; Whang et al., 2020).

Toll-like receptor agonists for all the existing receptors except TLR1 and TLR6 have been tested in preclinical tumor models. The agonists were either added to NPs (liposomes, PLGA, pH-sensitive, CO_2_ releasing), peptide nanocomplexes, or forming immunoconjugates with other antibodies. NPs were designed to preserve the agonist, target specific cells within the TIME and release their immunostimulant cargo. These NP-based therapies mainly targeted TAMs and APCs, aiming to reduce the regulatory phenotype of TAMs and induce presentation and co-stimulatory molecules on APCs. Other studies have demonstrated that targeting cancer cells with NPs delivering agonists for TLR3 induces direct apoptosis of mutated cells (Schau et al., 2019).

Of interest, a preclinical combinatorial approach of NP-loaded TLR agonists with other already-approved immunotherapies such as immune checkpoints or EGFR blocking antibodies led to a more significant antitumor immune response and prolonged survival. These results were associated with an increased count of M1 macrophages in the tumor and a better safety profile of the treatments (Paone et al., 2008; Han et al., 2016; Rodell et al., 2018; Buss and Bhatia, 2020; Chuang et al., 2020; Kim et al., 2020; Smith et al., 2020). Notably, some therapeutic approaches even induced an abscopal effect on distant tumors (Buss and Bhatia, 2020).

Overall, TLR agonists in clinical settings have demonstrated antitumor effects when combined with immunotherapies, but, as monotherapy, their efficacy remains limited (Frega et al., 2020; Le Naour et al., 2020).

#### The Tumor Necrosis Factor Receptor Superfamily

The tumor necrosis factor receptor (TNFR) superfamily is a class of receptors involved in several T cell activities. In particular, the engagement of these receptors determines the frequency of effector and memory T cells in response to antigen stimulation (Croft, 2009; Bremer et al., 2013). Indeed, these receptors can activate both innate and adaptive branches of the immune system, promoting the production of cytokines such as IL-4 and IFN-γ by lymphocytes and IL-1 and IL-12 by professional or non-professional APCs (Croft, 2009; Bremer et al., 2013).

It is known that TNFRs deliver co-stimulatory signals, as their effects largely depend on antigen recognition and TCR signaling (Croft, 2009). Thanks to their co-stimulatory signals, activating antibodies against TNFR family members 41BB, CD40, OX-40 have been tested in combination with antibody therapies against EGFR and immune checkpoints using NPs (especially PLGA NPs and carbon nanotubes) or nanocomplexes. The combination therapies resulted in more robust antitumor immune responses than each monotherapy, suggesting that polytherapy represents a better option for promoting a hot tumor (Dominguez and Lustgarten, 2010; Cruz et al., 2014). To further improve TNFR superfamily based therapies, NPs/nanoengagers have been functionalized with specific antibodies to promote activation of DCs and NK cells. The NPs carrying the stimulating cargo contributed to developing an immunogenic hot TIME eliciting a robust antitumor response (Dominguez and Lustgarten, 2010; Cruz et al., 2014; Au et al., 2020) in the presence of neglectable induction of regulatory T cells (Au et al., 2020).

#### cGAS-STING Pathway Activation

Cytosolic exogenous or endogenous DNA represents a danger signal for cells. The cyclic guanosine monophosphate (GMP)-adenosine monophosphate (AMP) synthase (cGAS) rapidly detects the cytosolic DNA to mount an innate response through the activation of the stimulator of interferon genes (STING). STING activation has been investigated as a co-stimulatory strategy to boost the immune response against cancer by mediating IFN type I responses in both innate and adaptive immune systems and by activating cell death in some cancer cells (Larkin et al., 2017; Liu and Guan, 2018; Bishop et al., 2020). Scientists hypothesized that targeted delivery of STING agonists such as cyclic dinucleotide (CDN) could potentially activate immune-suppressed tumors, turning them from cold to hot. For this purpose, agonists (Wilson et al., 2016; Cheng et al., 2018; Liang et al., 2020; Park et al., 2020), polymers directly inducing STING activation (Luo et al., 2017; Li et al., 2021) or chemotherapies inducing cytosolic DNA breaks (Eldehna et al., 2020) have been loaded on NPs (e.g., nanocages, liposomes, polymers, and hydrogels) designed to specifically target DCs and macrophages, demonstrating efficacy in preclinical tumor models. The use of liposome and silica NPs improved STING agonist responses, promoting a better infiltration of lymphocytes and reprogramming TAMs toward an M1 phenotype in murine tumor models (Cheng et al., 2018). Strikingly, a newly synthesized STING-activating polymer induced a prolonged activation (Li et al., 2021) and led to a robust antitumor immunity when combined with an antigen (Luo et al., 2017). The antigen-specific tumor response was even more remarkable when the STING-activating polymer was combined with conventional ligands (Li et al., 2021) or radiotherapy (Luo et al., 2019). Similarly, inhalable NPs loaded with a STING agonist combined with radiotherapy-induced systemic anticancer immunity with control over metastases (Liu et al., 2019). Effects of STING activation were also evaluated in combination with chemotherapeutic drugs or ICB, showing that STING-activating NPs modulate the TIME to promote responses of cold tumors insensitive to conventional monotherapies (Wilson et al., 2016; Liang et al., 2020). Of note, at the best of our knowledge, STING activating nanotechnological have not reached the readiness level for clinical trials.

#### Cytokines

Cytokines represent soluble mediators of cell communication and play a fundamental role in several pathophysiological conditions. The strategic modulation of cytokine secretion, circulation, and binding to their dedicated receptors is a successful approach to treating several diseases (Bird, 2016; Rider et al., 2016). In a melanoma mouse model, Hong et al. (2015) showed that PLGA particles loaded with IL-15/IL-15R complex were internalized by DCs and allowed *trans-*presentation of IL-15 to lymphocytes resulting in their activation and related enhancement of antitumor immune response.

Ramesh et al. (2019) focused instead on the functions of CSF1R in TAMs differentiation. An amphiphilic inhibitor of CSF1R was loaded into liposomes together with an inhibitor of the immune checkpoint CD47-SIRPα, adopted by cancer cells to escape from immune-mediated clearance. The administration of this dual therapy in a mouse model increased the percentage of M1-like macrophages and raised the infiltration of T cells and NK cells within the tumor, leading to a significant reduction in the tumor volume (Ramesh et al., 2019).

Cell membrane-adhering NPs loaded with cytokines have been used as a “cellular backpack” to sustain activation of cells after adoptive transfer in *in vivo* preclinical models. IFN-γ loaded backpacks binding to macrophages continuously guided the polarization of macrophages toward antitumor phenotypes (Wyatt Shields et al., 2020). Similarly, a backpack loaded with an IL-15 super-agonist increased T cell-mediated tumor clearance by mouse CD8^+^ T cells and human chimeric antigen receptor (CAR)-T cell therapy in a mouse model (Tang et al., 2018).

As demonstrated by these promising studies, targeting cytokine signaling with NPs represents a valuable strategy to turn a cold tumor hot.

### NP-Mediated DNA and RNA Targeting

Gene silencing technologies, namely CRISPR-Cas9 and RNA interfering (RNAi), have gained attention as potential therapeutic tools in many diseases, including cancer (Mainini et al., 2020; Chen et al., 2021). To achieve cell-targeted delivery of nucleotide sequences *in vivo*, NPs, either organic or inorganic, are indispensable. In principle, DNA and mRNA targeting applies to any possible cellular target mentioned in the previous sections to modulate cold TIMEs. Small interfering RNA (siRNA) and short hairpin RNA (shRNA) are also applied in the modification of the TIME from cold to hot, as shown by the administration of siRNA/shRNA/miRNA encapsulated in various NPs that successfully reduced the expression of key proteins in cancer cells, endothelial cells, TAMs, DCs, monocytes and CAFs (Lee S. W. L. et al., 2019; Roscigno et al., 2020). For example, in different preclinical tumor models, CRISPR/Cas9 knockout or siRNA/shRNA/miRNA-mediated silencing of PD-L1 immune checkpoint, in combination with chemotherapy, hyaluronidase, or radiotherapy, promoted an increased T cells activation and tumor infiltration (Cortez et al., 2016; Guan et al., 2019; Wu et al., 2019; Xue et al., 2021). In two murine models of colon cancer, Liu et al. demonstrated the antitumor effects of IL-15, performing an *in vivo* transfection of cancer cells with an IL-15 plasmid contained in lipoplexes. By increasing the tumor expression of IL-15, the tumor acquired hot features with a raised infiltration of IFN^+^ T cells (Liu et al., 2018). Similar siRNA-mediated silencing approaches targeting lactic dehydrogenase of cancer cells (Zhang et al., 2019), VEGFR of endothelial cells (Cho et al., 2020), indoleamine 2,3-dioxygenase (IDO) of DCs (Endo et al., 2019) showed promising results in modulating tumors toward hot features. Interestingly, in preclinical tumor models, siRNA-regulated expression of STAT3, STAT6, IKKβ HIF-1α proteins for TAM differentiation (Shobaki et al., 2020; Xiao et al., 2020), and of CCR2 receptor for monocyte recruitment (Shen et al., 2018) enriched the TIME of antitumor M1-like macrophages and reduced the number of immunosuppressive cells. Inhibition of CXCL12 in CAFs using siRNA-loaded into a cell-penetrating peptide (CPP)-based NPs induced remodeling of TIME inhibiting cell invasion, migration, and tumor angiogenesis (Lang et al., 2019). Both RNA and DNA editing techniques have already reached clinical trials, and their applications for turning a cold tumor hot may reach the clinical phases in the next future.

### NP-Delivered Small Molecules Inducing Cellular Stress, Immunogenic Cell Death, and Epigenetic Modifications

Cellular stress and immunogenic cell death (ICD) promote intra- and extracellular danger signals that mediate the activation of inflammatory pathways involved in the onset and maintenance of antitumor responses. Several molecules for cancer treatment can induce cellular stress (e.g., increased ROS and DNA damage) that can be sensed by immunocompetent cells, such as macrophages and DCs, sustaining inflammation and antigen presentation, thus, in turn, promoting a hot TIME. NP-targeted delivery of well-known chemotherapeutic drugs (e.g., intercalating agents and PARP1 inhibitors) or NP-assisted photothermal and radiotherapies provide *in situ* release of endogenous antigens and DAMPs, to boost antitumoral responses, especially if combined with ICB and to lower the systemic toxicity (Gao et al., 2020; Qi J. et al., 2021). DAMPs released by stressed cells promote activation of macrophages and their reprogramming toward an M1 proinflammatory phenotype. TAMs reprogramming toward an M1 phenotype is also promoted *in vitro* by NP-delivered compounds causing cellular stress such as low concentration of zoledronate loaded into liposomes (Sousa et al., 2015). Alternatively, TAM depletion is achieved by treatment with liposomes loaded with a high concentration of bisphosphonate that causes macrophage apoptosis (Zeisberger et al., 2006). TAMs depletion could be associated with an inflammatory response with increased T cell infiltration within the tumor, as shown in a mammary tumor animal model (Hollmén et al., 2019). Other TAMs depletion studies demonstrated their role in promoting tumor growth via sustained chronic inflammation, favoring angiogenesis, and establishing an immunosuppressive environment. On the other side, depleting TAMs rules out the possibility of leveraging the antitumor function of macrophages to have a synergistic effect with T cells (Poh and Ernst, 2018; Hollmén et al., 2019). Interesting, even macrophage phagocytosis of empty iron oxide or cationic polymer NPs (without a biologically active cargo) increased proinflammatory cytokines and immune cell infiltration, modulating the TIME toward hot characteristics (Lunov et al., 2011; Huang et al., 2013; Fuchs et al., 2016; Zanganeh et al., 2016).

Scientific evidence in favor of autophagy manipulation as cancer treatment is promising but sometimes contradictory. Studies showing iron oxide nanoparticle-mediated activation of tumoricidal autophagy in cancer cells and M1 polarization of macrophages (Xie et al., 2020) are in contrast to other studies demonstrating improved therapeutic effects of a combination of autophagy inhibitors (e.g., hydroxychloroquine) and antineoplastic therapies in clinical trials (Jiang et al., 2019) or inflammatory features in macrophages after autophagy inhibition (Jin et al., 2019).

Epigenetic modulators are another category of molecules that might benefit from the development of NP-based delivery strategies. For example, histone deacetylase inhibitors (HDACi) have been implemented within tumor-targeting particles showing significant inhibitory effects on tumor growth (Bertrand et al., 2019; Lee S. Y. et al., 2019). Studies evaluating the effects on the TIME of targeted HDACi therapy should be encouraged after the effects observed by selective HDACi in an NP-free treatment (McCaw et al., 2019).

## Discussion

The recognition of the TIME predominant role in mediating the effectiveness of chemotherapies and immunotherapies has propelled the development of new strategies to overcome tumor resistance by turning immunologically cold tumors into hot ones. NPs have greatly helped this purpose as nanocarriers of chemical and biological factors, improving their targeted delivery, accumulation, and retention at the tumor site. However, it is a long way to demonstrate the clinical reliability of novel NP-based therapeutic approaches to regulate the immune processes within the TIME. With the support of recent nanotechnologies, the immunotherapy field might aim to identify new applications of pre-validated drugs and their combinations. For example, marketed drugs called statins primarily used against hypercholesterolemia were re-purposed to treat inflammatory and immune-mediated conditions when loaded on NPs that reduced their marked myotoxicity (Romana et al., 2014; Sonvico et al., 2017). Similarly, drugs previously excluded during the R&D stage due to unwanted toxicity might find suitable applications when administered by NPs (Goldman et al., 2016; Chen et al., 2021). In addition to therapies directly affecting stromal cells, future efforts could be spent on developing NP-based therapies capable of tuning tumor-cell intrinsic pathways such as mTOR, p53, or STAT3 in cancer cells for the indirect modulation of the tumor immune microenvironment. Indeed, recent studies underlined that tumor-intrinsic pathways are involved in the regulation of the TIME and may support a tumor immunosuppressive microenvironment (Yang et al., 2019). Importantly, personalized polytherapy approaches guided by a prompt TIME classification should be prioritized to provide improved clinical benefits to each specific cancer patient.

## Author Contributions

GG and GA: conceptualization. GG: drafting. GA and AP: editing and revision. AP: figure. GG and GA: figure revision. All authors contributed to the article and approved the submitted version.

## Conflict of Interest

The authors declare that the research was conducted in the absence of any commercial or financial relationships that could be construed as a potential conflict of interest.
